# A partnership between academic and public librarians: “What the Health” workshop series

**DOI:** 10.5195/jmla.2019.564

**Published:** 2019-04-01

**Authors:** Jessica A. Koos, Jamie Saragossi, Gregg A. Stevens, Salvatore Filosa

**Affiliations:** Health Sciences Librarian, Health Sciences Library, Stony Brook University, Stony Brook, NY, jessica.koos@stonybrook.edu; Head, Health Sciences Library, Stony Brook University, Stony Brook, NY, jamie.saragossi@stonybrook.edu; Health Sciences Librarian, Health Sciences Library, Stony Brook University, Stony Brook, NY, gregg.stevens@stonybrook.edu; Marketing and Outreach Librarian, Port Jefferson Free Library, Port Jefferson, NY, sfilosa@portjefflibrary.org

## Abstract

**Background:**

Public librarians are in a unique position to assist the general public with health information inquiries. However, public librarians might not have the training, detailed knowledge, and confidence to provide high-quality health information.

**Case Presentation:**

The authors created and delivered three workshops to public librarians in Suffolk County, New York, highlighting several National Library of Medicine resources. Each workshop focused on a different topic: general consumer health resources, genetics health resources, and environmental/toxicology resources. At the end of each workshop, participants were asked to complete the Training Session Evaluation form provided by the National Network of Libraries of Medicine (NNLM). All participants reported that they learned a new skill or about a new tool, that their ability to locate online health information improved, and that they planned to use the knowledge they gained in the future. Online tutorials covering the major resources from each workshop were created and made accessible to the public on several organizations’ websites. Virtual reference services were initiated for public librarians who need further assistance with these resources and will continue to be provided on an ongoing basis. Financial support for the equipment and software utilized in each of these tasks was awarded by NNLM.

**Conclusions:**

Based on attendance and participant feedback, this model of health information outreach appears to have been successful in furthering the educational needs of public librarians and may be useful to others in creating a similar program in their communities.

## BACKGROUND

The National Action Plan to Improve Health Literacy, published in 2010, focuses on the importance of partnerships and a “multisector effort” in achieving the goal of better health outcomes through increased health literacy. One of the basic principles upon which this plan was founded is that “all people have the right to health information that helps them make informed decisions” [1]. Academic health sciences librarians are uniquely positioned to assist in this effort by providing training, access to resources, and the skills necessary to evaluate health information. Workshops can be offered to the public as well as to public librarians, who often encounter initial health-related reference questions.

Many consumers tend to gravitate to public libraries to obtain general health information or to locate additional information about a recent diagnosis or treatment. According to Yi, public libraries are often viewed as the most accessible and inexpensive means of obtaining information, including health information [2]. Luo and Park indicate that human-mediated reference that is provided at public libraries can assist in successfully retrieving the information being sought [3]. However, this can be challenging for public libraries for many reasons. For example, public libraries have such a broad scope of content and services that they are unable to collect deeply in the area of medical or health information due to both budgetary and space constraints [4].

Research into the self-perceived consumer health knowledge gaps of public librarians shows that they would like to know more about credible health information that patrons can easily understand [3]. Clifton and colleagues highlight the importance of academic health sciences librarians collaborating with public librarians, acknowledging that academic librarians must always keep in mind the importance of supporting public librarians in their mission to provide the best possible programs and services to their patrons. This can be more readily achieved by creating relationships and continuing to build these encounters into partnerships that actively address communities’ common concerns [5].

## STUDY PURPOSE

Previous studies show that public librarians can improve their knowledge of consumer health resources through instruction from academic health sciences librarians [5–7]. Based on this evidence, the authors implemented a project in which academic health sciences librarians educated and provided support to public librarians in Suffolk County, New York, about the use of freely available, reliable online health resources, with a primary focus on National Library of Medicine (NLM) databases.

## CASE PRESENTATION

Stony Brook University is a public research university located in Suffolk County, New York. Believing that they could contribute to the overall health of their community, librarians at the Stony Brook University Health Sciences Library proposed a “Health Information Awareness Outreach” project. Suffolk County occupies the eastern end of Long Island, with a total population estimated at 1.5 million with a median age of 41.9 years. Of those between 18 and 64 years of age, 14.5% are uninsured, meaning that health care and quality health information may not always be available or accessible [8]. In addition, while Suffolk County may be considered an affluent area due to having a median household income of $90,128 per year, an income disparity still exists, with 7.6% of the population living in poverty [9]. These factors provided evidence that this community could benefit from enhanced consumer health services at public libraries.

This project consisted of three main components: in-person workshops, online tutorials, and virtual face-to-face support for Suffolk County’s public librarians. Funding to support this initiative was received from the National Network of Libraries of Medicine (NNLM), Middle Atlantic Region, through its Health Information Awareness Award. This award is designed to support “small outreach projects that increase awareness of NLM products” as well as health information services provided by various types of organizations [10]. The funds were used to purchase ten iPads to be used during the workshops, one license of Camtasia screen capture software to create online tutorials, and three webcams to provide virtual support to librarians or patrons who required additional one-on-one support.

The “train the trainer”-style series of workshops, titled “What the Health,” was promoted through various outlets; however, efforts were focused on obtaining buy-in from committee chairpersons of the Reference and Adult Services Division (RASD) of the Suffolk County Library Association (SCLA). RASD is the largest division of SCLA and hosts seventeen committees relating to the continuing education of public reference librarians in Suffolk County. The committee chairpersons shared the information about these workshops in a more organic way that might have resulted in higher attendance than solely utilizing formal communications.

To reach other public librarians in Suffolk County, print and digital fliers were created and shared on email discussion lists for SCLA members, forums for the Suffolk Cooperative Library System (SCLS) member librarians, and social media pages for RASD followers. Students of library science were also invited to attend all three workshops through library school email discussion lists and library student groups on Facebook. Members of other local organizations, such as the Long Island Library Resource Council’s Health Sciences Information Committee and the Medical and Scientific Libraries of Long Island, were also invited to attend and were encouraged to promote these workshops. A partnership with the administrator of outreach services at SCLS was created to secure use of training facilities, which included an integrated podium with a computer, projector, overhead speakers, and twenty-four computer workstations.

Three two-hour workshops were provided, with each covering a different topic. Each in-person workshop was taught by a different Stony Brook health sciences librarian, based on their subject knowledge and expertise. Participants could register for one, two, or all three workshops. Handouts containing the web addresses and brief descriptions of resources were provided.

Toward the end of each class, hands-on exercises were distributed for completion. The participants worked alone or in small groups to complete the exercises. iPads were distributed to the participants, who were encouraged to use them as a way of learning how to use the mobile versions of the NLM websites. The instructor then reviewed the activity with the class as a whole to discuss possible search strategies as well as differences in mobile versus web-based access, allowing immediate feedback and informal learning assessment. The iPads also served as a backup in case there were not enough classroom computers available for attendees.

The first workshop focused on NLM’s MedlinePlus, which is considered the National Institutes of Health (NIH) primary database for patients, caregivers, and family members. This resource was selected for the first workshop to establish a baseline understanding of health literacy and an understanding of a general consumer health resource that encompasses a broad range of health topics and demographics. A focus of this workshop was to provide scenarios that mimic actual health reference questions, followed by hands-on searching and discussion amongst the participants. The goal of this activity was not only to share how participants approached the search, but also to have the group communicate about health topics and feel more comfortable conducting reference interviews and discussions with patrons who inquire about health-related topics.

According to the US Census Bureau, Spanish is the second most common language spoken in Suffolk County, New York, and Latinos make up more than 15% of Long Island’s population [11]. Because of this, it was important to show that NLM provides resources for Spanish language translation of the website as well as handouts and topic searches in Spanish and many other languages. Reviewing these features that are available in MedlinePlus allowed the instructor to facilitate a discussion with participants on the importance of health literacy, cultural competence, and reading level when providing health information.

The second workshop covered genetic health resources, with the main focus on the NLM Genetics Home Reference database. With the recent implementation of NIH’s “All of Us” project, the popularity of genetics home testing kits, and recent innovations in genetic treatments for certain conditions, we surmised that public librarians could receive a significant amount of reference inquiries on such topics. The presentation and handout were adapted from a similar course, ABCs of DNA: Unraveling the Mystery of Genetics Information for Consumers, created by Terri Ottosen, AHIP, formerly of the Southeastern/Atlantic Region of NNLM. The class began with a brief overview of genetics in the news and popular culture to highlight the relevance of being able to locate genetic health information. Since the topic of genetics can be somewhat complex, a review of basic genetic components was also provided. In addition to providing guidance on navigating Genetics Home Reference, other resources were also discussed, including the National Organization for Rare Disorders website.

The topic of the third workshop was resources for environmental health and toxicology, with a primary focus on TOXNET, an NLM suite of databases. Because many TOXNET databases are mainly of use to researchers and health care professionals, the instructor chose to introduce the participants to TOXNET’s environmental health portal and two of its databases with relevance to consumer health inquiries: the Household Products Database and TOXMAP. Additionally, the instructor introduced attendees to a related resource outside of the TOXNET suite, Tox Town. Presenting these resources anticipated that environmental health questions might be received in a public library, such as how to find information on potential local environmental threats due to toxic chemical releases. The instructor also consulted with staff from NNLM’s National Training Office regarding the selection of the databases to be presented.

Tutorials focusing on the main resources that were covered in each workshop were created using Camtasia. Each tutorial was approximately two to three minutes long. At the conclusion of the workshops, the online tutorials were shared and posted to various topic guides and forums hosted through the SCLS, RASD, and Stony Brook University Health Sciences Library. Because these tutorials were introductory in nature, they were made fully accessible to all Suffolk County librarians, library school students, and the general public.

Virtual face-to-face reference services were also made available on an ongoing basis to public librarians in case they required additional support after the workshops had taken place. Assistance is provided to the public librarians themselves and any patrons they refer to academic librarians for further consultation. These services are provided by Stony Brook health sciences librarians utilizing webcams to facilitate communication and timely assistance. Software such as Google Hangouts is utilized to allow individuals to view one another during a consultation and to share computer screens to aid in instruction and troubleshooting. As Stony Brook is a public university, this service is congruent with its mission of serving the public and will continue to be made available into the future beyond the scope of this project.

A total of 27 different librarians attended at least 1 class in the workshop series. These librarians came from 20 different public library branches across Suffolk County, representing nearly one-third of the SCLS branches. The average librarian attended more than 1 workshop (mean=1.63 workshops, standard deviation [SD]=0.688). The MedlinePlus workshop had 17 participants, the Genetics Home Reference workshop had 15 participants, and the TOXNET workshop had 13 participants. The TOXNET workshop attendance might have been lower because it was postponed due to a winter storm on the original class date and rescheduled for 2 weeks later. Because of the short notice for the new date, some librarians who originally signed up for this class were not able to attend on the make-up date.

Formal assessment was conducted using the NNLM Training Session Evaluation form, which uses five objective questions to determine the efficacy of the session. This form was used by the authors as an assessment tool as it was required by the NNLM award. The questions on the form are designed to elicit whether the participants learned at least one new skill or about a new tool in the session and whether their general ability to locate health information improved. The form also includes questions about the participant’s plans for using this information in the future.

Of the 45 evaluation forms that were received, all (100%) indicated that the participant was introduced to a new resource. When asked if the participant planned to use a new skill in the future, almost all (n=44) indicated “Yes,” with one indicating “Not Applicable.” For the statement, “This training improved my ability to find useful online health information,” 1 attendee from the MedlinePlus workshop indicated “Somewhat Agree,” whereas all others (n=44) indicated “Strongly Agree.” For the statement, “I plan to start using at least one resource or tool that I learned about in this training,” 1 participant from the genetics workshop indicated “Somewhat Agree,” another from that workshop indicated, “Somewhat Disagree,” and all others (n=43) indicated “Strongly Agree.” For the final statement, “I plan to tell others about at least one resource or tool that I learned about in this training,” the same 2 participants from the genetics workshop who indicated “Somewhat Agree” or “Somewhat Disagree” with the previous statement again indicated “Somewhat Agree,” and all others (n=43) indicated “Strongly Agree.”

There was also a substantial amount of participant engagement during the workshops, which provided an informal indication of attendee satisfaction. Some public librarians shared specific cases and questions that they received at their branches that could have been answered with the presented resources. For example, during the environmental health workshop, a few librarians discussed how their branches housed print records about local Superfund sites. They stated that these materials were often requested for viewing by patrons, and they were pleased to know that much of that information is also available online through TOXMAP. Additionally, many librarians cited a need for consumer health materials for patrons for whom English is not their first language. These librarians were impressed by the number of foreign language materials available on MedlinePlus.

## DISCUSSION

As previously stated in the literature, academic health sciences librarians are uniquely positioned to address the need for educational opportunities in the area of consumer health [5–7]. As Luo and Park demonstrated in their 2013 study, public librarians desire additional educational opportunities in several key areas: knowledge and navigation of core health resources, health reference interviews, and health references for specific populations such as low literacy and non-English speakers [3]. It was essential when developing and delivering all “What the Health” workshops that we addressed these top three concerns.

The number of workshop attendees, participant engagement, and survey responses provide strong evidence that this project has been successful in meeting the health information needs of public librarians. This program contributes to the body of evidence supporting the idea that academic health sciences librarians can successfully provide instruction to public librarians in the area of consumer health. It also demonstrates a successful modality that can serve as an example for other academic librarians who are interested in creating similar programs. The Medical Library Association (MLA) has acknowledged the importance of partnering with public librarians by holding a “Health Information for Public Librarians” symposium at its 2018 MLA annual meeting. The symposium consisted of informational sessions designed to teach librarians about consumer health resources, inform them of NLM initiatives, and provide opportunities to network with health sciences librarians [12].

Several librarians who attended the workshops stated that they wanted to run similar programs for patrons but were not previously comfortable enough with the resources to conduct them. Therefore, these workshops equipped public librarians with the knowledge and confidence to educate community members about consumer health resources. This ripple effect of information hopefully will spread and amplify the information that was conveyed well beyond the confines of the three workshops. Various committee chairpersons for RASD indicated that all links from the handouts will be added to a resource page or topic guide on their library websites. As evidenced by feedback from participants, the main goal of the workshops—to equip public librarians with the proper consumer health knowledge to assist library users—was reached.

There were several important “lessons learned” from this project. First, it is essential to work with an individual from the public library sector in all stages of the planning process. This was crucial to facilitate navigating the public library system and its affiliated organizations. It also provided the opportunity for promoting this program in the SCLS. Second, the time of year that workshops are offered can negatively impact attendance due to the occurrence of inclement weather, even when they are rescheduled. As mentioned previously, when one of the workshops was rescheduled due to a winter storm, the attendance markedly declined from the number that had originally registered.

As previously discussed, projects in which academic health sciences librarians provide training to public librarians are not unique [5–7]. However, there are several factors that set this project apart from the others. Unlike these other projects, this one had a very narrow focus, as it involved only a single public library system. This project is only the first phase of developing a long-term relationship where one had previously not existed. The collaborative reference assistance on health inquiries via webcams will continue to be offered on an ongoing basis. This affords a unique opportunity for public librarians to have direct contact to support their health reference transactions and assistance to ensure they are providing access to high-quality consumer health information. Thus, this project provides evidence of a novel way to create and maintain a relationship between an academic library and a public library system.

In addition, the types of content covered in each workshop in this project were also distinct. While MedlinePlus is a primary consumer health information resource, the other topics of genetics and environmental health create a unique combination of subject areas. The participants’ appreciation for and perception of the utility of these topics provides evidence that they might be beneficial to address in future offerings to public librarians.

The resources developed in this project—including the workshop presentation files, handouts, and tutorials—are freely available to librarians in other communities who wish to pursue a similar project, through the Consumer Health: What the Health research guide on the Stony Brook Health Sciences Library website. We hope that continuing this project across different communities will help to reduce health disparities and improve the health and well-being of countless individuals.
